# Cationic cellulose filter papers modified with ZnO/Ag/GO nanocomposite as point of use gravity-driven filters for bacterial removal from water

**DOI:** 10.1038/s41598-023-50114-3

**Published:** 2023-12-18

**Authors:** Seyed-Behnam Ghaffari, Mohammad-Hossein Sarrafzadeh

**Affiliations:** UNESCO Chair on Water Reuse, School of Chemical Engineering, College of Engineering, University of Tehran, P.O. Box 11155-4563, Tehran, Iran

**Keywords:** Antimicrobials, Graphene, Nanoparticles, Chemical engineering

## Abstract

The surface modification of filters with large pore sizes for the development of low-cost gravity-driven point-of-use (POU) technologies for water disinfection can be an effective strategy to empower people to access safe water instantly, especially in low- and middle-income countries. In this study, the surface of commercial cellulose filter papers, as cheap and bio-based filters, was modified with polydopamine (PDA), polyethyleneimine (PEI) and ZnO/Ag/GO nanocomposite (ZnO/Ag/GO@PDA/PEI papers) for bacterial removal from water. PDA/PEI incorporation introduced a cationic functional layer, which can entrap negative bacteria and make a stable chemical bond with the nanocomposite. ZnO/Ag/GO exhibited promising synergistic antibacterial activities (30 times stronger than ZnO). As a result, 3 sheets of ZnO/Ag/GO@PDA/PEI papers showed a 99.98% bacterial reduction (*E. coli*), which met the WHO standards. Moreover, the leached zinc and silver in the filtrate were far below the WHO’s limits (380 and 10 ppb, respectively). The results showed that the modified papers could be reused multiple times. After six times of reuse, the flow rate dropped slightly (below 20%) and the bacterial removal efficiency was more than 99.9%. This study is valuable for developing filters for treating bacterial-contaminated water on-site with no need for energy, which is a demand in many countries.

## Introduction

Access to safe water is considered a human right, and efforts led to the improvement of the proportion of the world's population with access to safe water from 76% in 1990 to 91% in 2015^1^. However, huge gaps remain. About 800 million people globally do not have access to basic safe drinking water and hygienic sanitation and use unimproved surface or groundwater sources^2^. Moreover, around 2 billion people were drinking water contaminated with feces, which contained pathogens^1^. Very low concentrations of pathogens in drinking water can be enough to cause harmful infections via ingestion, airborne or contact with water. Water-borne diseases are a significant public health concern, not only due to the outbreaks and mortality that they cause but also because of the effectiveness and costs of water disinfection methods^3^. Furthermore, it is estimated that waterborne illnesses have an economic cost of ~ 12 billion $ per year^4^. Therefore, there is an urgent demand for cost-effective production of high-quality drinking water, especially in low- and middle-income countries.

Various processes are used to remove and/or inactivate pathogens, with each process having its own benefits and disadvantages. The selection of the appropriate processes is based on different parameters: the kind and concentration of pathogens, the demanded effluent final quality, plant design and cost^5^. Conventional disinfection processes include using chemicals and UV light radiation. The use of chemicals such as chlorination and ozonation has been successful in protecting public health from waterborne diseases. However, there are serious concerns about the production of toxic byproducts such as ketones, aldehydes, and chlorate ions. On the other hand, high costs and high energy demand are drawbacks of UV radiation technology^6^. As a result, for years, researchers have sought to develop alternative methods. Low-cost and easy-to-use ones can be ideal candidates for developing portable POU technologies for drinking water disinfection as instant approaches for water treatment that empower people and communities without access to safe water to improve water quality by treating it at the point of consumption, including in homes, schools, and health centers^7,8^. In addition, when contaminated or disrupted water supplies occur in a natural disaster, people are left with no choice but to drink unsafe water^9^.

An advanced alternative to conventional disinfection methods is the filtration process. Membrane filtrations with a pore size of less than 10 nm, such as ultrafiltration (UF), nanofiltration (NF) and reverse osmosis (RO) can remove pathogens using size-exclusion^10^. However, the costs of these filtrations are high, and high flux values achieved by using high operation pressures were essential for making these systems economically feasible. On the other hand, filtration systems with large pores, such as microfiltration (MF), allow for high fluxes at low pressures, so they do not require electricity for operation and could be utilized by atmospheric pressure in household water treatment systems^11^.

The filters with large pores are designed specifically to separate large particles and microscopic, atomic or ionic materials, but small pathogens are still able to transfer through the pores. Therefore, they are rarely effective for pathogen removal without pre- or post-treatment procedures^10,12^. Bacteria can pass through filter pores, which are even smaller than their size due to deformation^13^. Therefore, it would be a real breakthrough if cheap gravity-driven filtration systems could be used as a POU technology to remove pathogens from surface water.

In recent years, the interest in the use of gravity-driven filters for water disinfection has increased^14^. For this aim, the surface modification of filters with a complex porous structure is an effective approach for sustainable pathogen reduction. Filter characteristics play a critical role in engineering interactions with bacteria to trap or deactivate them in a short time, which is generally referred to as “contact killing”^15^. Till now, three strategies have been performed to enhance the pathogen removal efficiency of the filters with large pores, including electrostatic repulsion or adsorption, hydrophobic interactions and very recently introducing antimicrobial nanoparticles^16^. The surface of filters with cationic layers provides an electrostatic adsorption approach to prevent the passage of negatively-charged microbes^17^. Furthermore, higher pathogen removal efficiency was observed with hydrophobic filters compared to hydrophilic ones^18^. Substantial efforts have been made to create antimicrobial surfaces using the antimicrobial potentials of nanoparticles such as Ag, Cu_2_O, TiO_2_, ZnO and carbon-based materials^15,19^. However, the use of nanoparticles incorporated into surface membranes was mostly investigated for reducing biofouling^20^. Recently, researchers used the incorporation of antimicrobial nanostructures into polymeric or ceramic membranes to attain better pathogen removal^21^.

As mentioned previously, for developing filter-based POU technologies, affordability is critical. Recently, cellulose filter papers with large pores, as cheap and bio-based POU filters, were subjected to instant water purification^22^. Various compounds, including cationic polymers, phenols, metals and very recently nanoparticles, were used for the modification of cellulose-based papers for bacterial removal^23^. Nevertheless, releasing compounds or nanoparticles from filters into water sources can have several environmental risks^24^.

In our preceding study, the surface of commercial cellulose filter papers was modified to develop cheap portable POU filters with the ability to instantly disinfect water with no need for energy. Two strategies were used for the surface modification of papers for pathogen removal while keeping the large pores. First, the polymerization of dopamine and polyethyleneimine for forming the electrostatic adsorption, and second, the incorporation of a ZnO/Ag/GO nanocomposite with strong antibacterial activity. Among various nanomaterials, ZnO exhibited promising potential for use in active layers of membranes because of its unique features, such as hydrophilicity, antimicrobial effect, photocatalytic activity and high active surface area. Moreover, ZnO is non-toxic and considerably cheaper than silver and other metal or metal oxide nanomaterials^25^. Keeping this in mind, a stable bond was made between the nanocomposite and the surface of the papers to avoid releasing the nanoparticles into the water. The papers were characterized, and their performance for bacterial removal was assessed. Based on the results, the engineered papers, which met WHO standards for drinking water, can be used as cheap gravity-driven filters to provide safe water for people several times.

## Methods

### Materials and chemicals

Whatman filter papers (N. 42, slow) with a reported mean pore size of 2.5 μm and a thickness of 0.39 mm were chosen as commercial cellulose filter papers. Zinc chloride, silver nitrate solution (0.1 M), sodium hydroxide, calcium hydroxide, ethylene glycol and dopamine hydrochloride were purchased from Merck Chemical Co. Polyethyleneimine (PEI, branched, MW 25,000 Da) was supplied by Sigma Aldrich Chemical Co. Graphene oxide (GO) sheets were bought from Platonic Nanotech Co.

### Synthesis of ZnO nanoparticles

1 M of zinc chloride solution (dissolved in 90 mL of 1:1 ethylene glycol/water) was prepared. Afterward, the solution was dropwise added to 80 mL of a 1 M Ca(OH)_2_ mixture (suspended in water). The mixture was refluxed for 2 h at 100 °C under continuous stirring. Finally, the obtained powder was separated by a centrifuge, washed several times with water and, then dried at 90 °C in an oven overnight. This sample was labeled as ZnO nanoparticles (NPs).

### Synthesis of ZnO/GO, and ZnO/Ag/GO nanocomposite

ZnO/GO nanocomposite was synthesized as reported by Mahlangu et al., with modifications^26^. 2 g of GO was exfoliated and dispersed in 100 mL distilled water using a bath sonicator for 40 min. Afterward, 2.7 g of zinc chloride and 40 mL of silver nitrate solution (0.1 M) were added to the mixture. The mixture was again sonicated for 40 min to make an appropriate contact between the GO, Zn and Ag salts. Then, sodium hydroxide solution (1M) was added drop by drop, under vigorous stirring, until pH = 11 was achieved. The mixture was stirred for one day at 80 °C. After separating using a centrifuge and washing particles several times with distilled water, they were dried at 70 °C in the air overnight. This sample was named ZnO/Ag/GO. The ZnO/GO sample was synthesized by the same procedure without adding the AgNO_3_ solution.

### Modification of filter papers

#### Preparation of PDA and PDA/PEI modified filter papers

For the surface modification of filter papers, they were soaked in a reaction solution at room temperature for 2 h under constant shaking. In order to prepare the PDA modified filter papers, the reaction solution consisted of dopamine (2 mg/mL) dissolved in Tris buffer (pH = 8.5). For PDA/PEI modification of the papers, PEI (1 mg/mL) was also added to the Tris buffer. Afterward, the papers were washed with distilled water several times and dried in the air.

#### Preparation of ZnO/Ag/GO incorporated PDA/PEI modified filter papers (ZnO/Ag/GO@PDA/PEI papers)

For incorporation of as-synthesized ZnO/Ag/GO nanocomposites into the surface of filter papers, 20 mg of ZnO/Ag/GO particles were added to the Tris buffer and then dispersed by 30 min sonication. Next, dopamine and PEI were added to the mixture, similar to the process described in “Preparation of PDA and PDA/PEI modified filter papers” section.. Next, filter papers were soaked in the prepared mixture for 2 h at room temperature under constant shaking. Finally, papers were rinsed with distilled water several times and dried in the air. These papers were labeled as ZnO/Ag/GO@PDA/PEI papers.

### Characterization of the products

XRD (X-ray diffractometer; Panalytical, X’ Pert Pro, using Cu K_α1_ radiation) analysis was performed to evaluate the phase characterization of the nanostructures. FTIR (Fourier Transform Infrared Spectrophotometer; Thermo Avatar) was used through KBr pellets in the region between 400 and 4000 cm^-1^ to study the chemical structure of the powders, while for the filter papers, ATR (the variable angle attenuated total reflectance) mode was taken in the region between 600 and 4000 cm^-1^. The morphology of the particles and filters was imaged using a field-emission scanning electron microscopy equipped with an EDS energy dispersive spectroscopy (FE-SEM; TESCAN MIRA 3) and a transmission electron microscope (TEM; Zeiss-EM10C-100 kV). The zeta potential of the filters was studied through an electrokinetic analyzer (SurPASS, Anton Paar Ltd, Austria) using a 1 mM KCl electrolyte solution. To evaluate the surface charge of the particles (dispersed in distilled water), a Horiba SZ-100 Zeta analyzer was performed. The UV–Vis diffuse reflectance spectroscopy (DRS) of the samples was recorded using a Scinco spectrophotometer (S-4100).

### Flow rate of the filter papers determination

The flow rate for filtration through paper was calculated by filtering distilled water through a specific number of sheets of paper. The filtration was carried out in the simplest way in which the papers (45 mm in diameter) were folded into a funnel shape and then put into a funnel. Before the test, the papers were washed with water for one minute. After the filtration of 50 mL of distilled water, the flow rate was reported as mL min^-1^ (the mean flow rate of three tests).

### Evaluation of nanostructures leaching into the filtered water

10 mL of distilled water was filtered with the modified papers in the way described in the former part. The concentration of Zn and Ag in the collected water was investigated using inductively coupled plasma mass spectrometry (ICP-MS, Agilent 7500).

### Antibacterial evaluation of nanostructures and the papers

#### Calculation of the minimum inhibitory concentration (MIC) and the minimum bactericidal concentration (MBC)

A broth susceptibility assay was performed to estimate the MIC and MBC values of the samples against *Staphylococcus aureus* (*S. aureus*) and *Escherichia coli* (*E. coli*), as gram-positive and gram-negative bacteria, respectively^27^. The assay tubes comprised one mL of various concentrations (from 1024 to 0.125 μg/ml) of each sample in Müeller Hinton (MH) broth solution, containing about 10^6^ cells. Afterward, the tubes were placed in a shaker incubator at 37 °C for 24 h. The lowest concentration at which the sample inhibited bacterial growth (the tube with no turbidity) was considered The MIC value. To calculate the MBC value, the tubes with no turbidity were cultured in MH broth. The MBC is the lowest concentration at which the sample killed 99.9% of the bacteria.

#### Disk diffusion assay of papers

The disk diffusion assay against *E. coli* was performed to test the antibacterial activity of the papers. First, disks with a diameter of 5 mm were prepared from the papers. Then, they were sterilized using ultraviolet. Freshly cultured bacteria (0.5 McFarland) were spread on MH agar by a sterile swap. Afterward, the disks were placed in the agar medium. Ampicillin (with a concentration of 3 μg per disc) and an empty disc were used as positive and negative controls, respectively. The pellets were incubated at 37 °C for 24 h. Finally, the inhibition zone diameter (mm) was measured by a ruler. The zone of inhibition (< 8 mm) was considered non-susceptible.

#### Bacterial removal efficiency of filtration

To investigate the bacterial removal efficiency of filter papers, after being folded into a funnel shape, they were put into a funnel. Filters were previously prepared by stacking a specific number of sheets of paper (45 mm in diameter) and washing them with water for one minute. The unmodified paper served as the control sample. The bacterial reduction efficiency was studied through the filtration of 10 mL of distilled water with a bacterial concentration of 1.5 × 10^8^ CFU/mL (*E. coli*). Then 1 mL of the permeate was separated, diluted and cultivated in plates to find the concentration of bacteria remaining (colony counting) in the permeate using ImageJ. The data presented are from three independent experiments.

### The assessment of the flow rate and the bacterial removal efficiency of filters in reuse

The performance of papers, including the flow rate and the bacterial removal efficiency when re-performing them, was studied by reusing them for six consecutive filtration procedures. For the assessment, three sheets of ZnO/Ag/GO@PDA/PEI papers were chosen. The procedures for the measurement of the flow rate and the bacterial removal efficiency were the same as the previously described methods.

## Statistical analysis

All data were collected in triplicate, and the statistical outcomes were evaluated by SPSS Statistics 22.0 and reported as mean ± standard deviation (SD).

## Results and discussion

In the current study, to produce a simple gravity-driven filtration system for water disinfection, filter papers were modified with cationic polymers and an antimicrobial nanocomposite. The engineering of the surface of the papers must result in the removal of bacteria upon of contact which is generally referred to as “contact killing”. To increase the contact killing efficiency of ZnO, a nanocomposite of ZnO and GO/Ag was developed.

### Structural characterizations of the nanostructures

**Figure 1:**
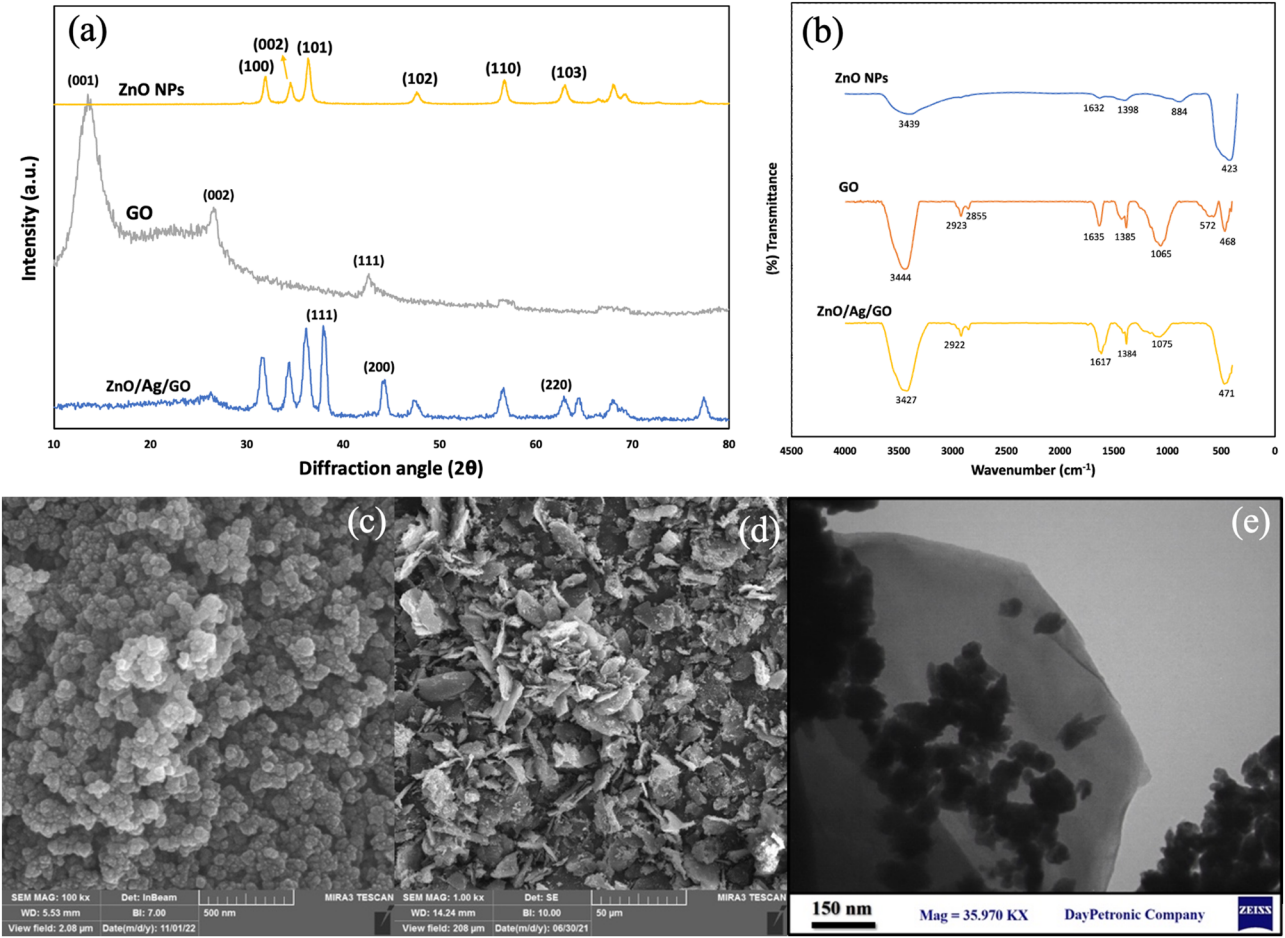
(**a**) XRD patterns of ZnO NPs, GO and ZnO/Ag/GO powders. (**b**) FTIR spectra of ZnO NPs, GO and ZnO/Ag/GO powders. (**c** and **d**) The FE-SEM image of ZnO NPs and ZnO/Ag/GO powders, respectively. (**e**) The TEM image of ZnO/Ag/GO powder.

XRD analysis was taken to confirm the formation of the target formulations. Figure 1a demonstrated the XRD patterns of ZnO NPs, GO sheets and ZnO/Ag/GO nanocomposites. In the case of ZnO NPs, all the diffraction peaks are related to the JCPDS file of hexagonal wurtzite zinc oxide (JCPDS #36–1451)^28^. No other peaks attributed to impurities were observed. Thus, the formation of ZnO crystals is verified. The formation mechanism of ZnO crystals from zinc-containing materials in chloride solutions was completely reviewed elsewhere^29^. The crystallographic planes corresponding to each peak are also indicated. In the XRD pattern of GO, three major peaks around 13°, 26° and 46°, indexing (001), (002) and (111) planes, respectively, can be seen. In the pattern of ZnO/Ag/GO, all peaks related to hexagonal wurtzite ZnO were present. However, the sharpest peak related to the (001) plane of GO disappeared, indicating the destruction and exfoliation of the regular layered structure of graphene oxide. In the synthesis procedure, zinc ions have bonded to the oxygen atoms of the functional groups on the surface of GO sheets. After adding the NaOH solution, the crystal growth units of ZnO crystals, such as Zn(OH)_4_^2−^ reacted with the mentioned functional groups, and therefore anchor sites for ZnO NPs were formed. Finally, numerous ZnO nuclei were formed by the hydrolysis of the growth units, and the ZnO/GO was produced^30^. In the diffraction pattern of ZnO/GO/Ag, the appearance of Ag-related peaks corresponding to (111), (200), and (220) crystallographic planes (JCPDS #04-0783) also confirmed the Ag formation.

FTIR spectroscopy as an analytical technique was performed to study the chemical structure of the as-synthesized products, and the spectra are shown in Fig. 1b. In the ZnO NPs spectrum, the sharp peak at 423 cm^-1^ is attributed to the Zn–O bond. The peak at 1398 cm^-1^ is due to the C–OH bond. Furthermore, a broad peak, presenting –OH groups, can be observed at 3439 cm^-1^^31^. In the FTIR spectrum of graphene oxide, the peak at 3444 cm^-1^ is attributed to the presence of the OH and groups^32^. Moreover, the peaks at 1635, 1385 and 1065 cm^-1^ are related to C=C, C–OH and C–O bonds, respectively^33^. In the FTIR spectrum of ZnO/Ag/GO, the intensity of the peak around 468 cm^−1^ amplified with shifting to 471 cm^−1^ because of the overlap with the peak associated with the Zn–O bond. Additionally, a significant decrease was observed in the intensity of the peak at 1065 cm^−1^, probably due to the bond between Zn or Ag with oxygen atoms on the surface of graphene oxide.

The chemical composition of the ZnO/Ag/GO sample was also evaluated using EDS analysis. The EDS characterization exhibited proportions of 29% zinc, 10% oxygen, 49% carbon, 5% nitrogen, and 7% silver.

The morphology of the samples was investigated using FE-SEM and TEM microscopes and images are shown in Fig. 1. Based on the FE-SEM image, ZnO NPs have a spherical-like shape and are uniform in shape and size, with a mean size of 40 nm. In the FE-SEM image of ZnO/Ag/GO powder, graphene oxide sheets are obvious. Sheets are decorated with spindle-like and spherical-like ZnO or Ag nanoparticles, which are better recognizable in the TEM image.

**Figure 2:**
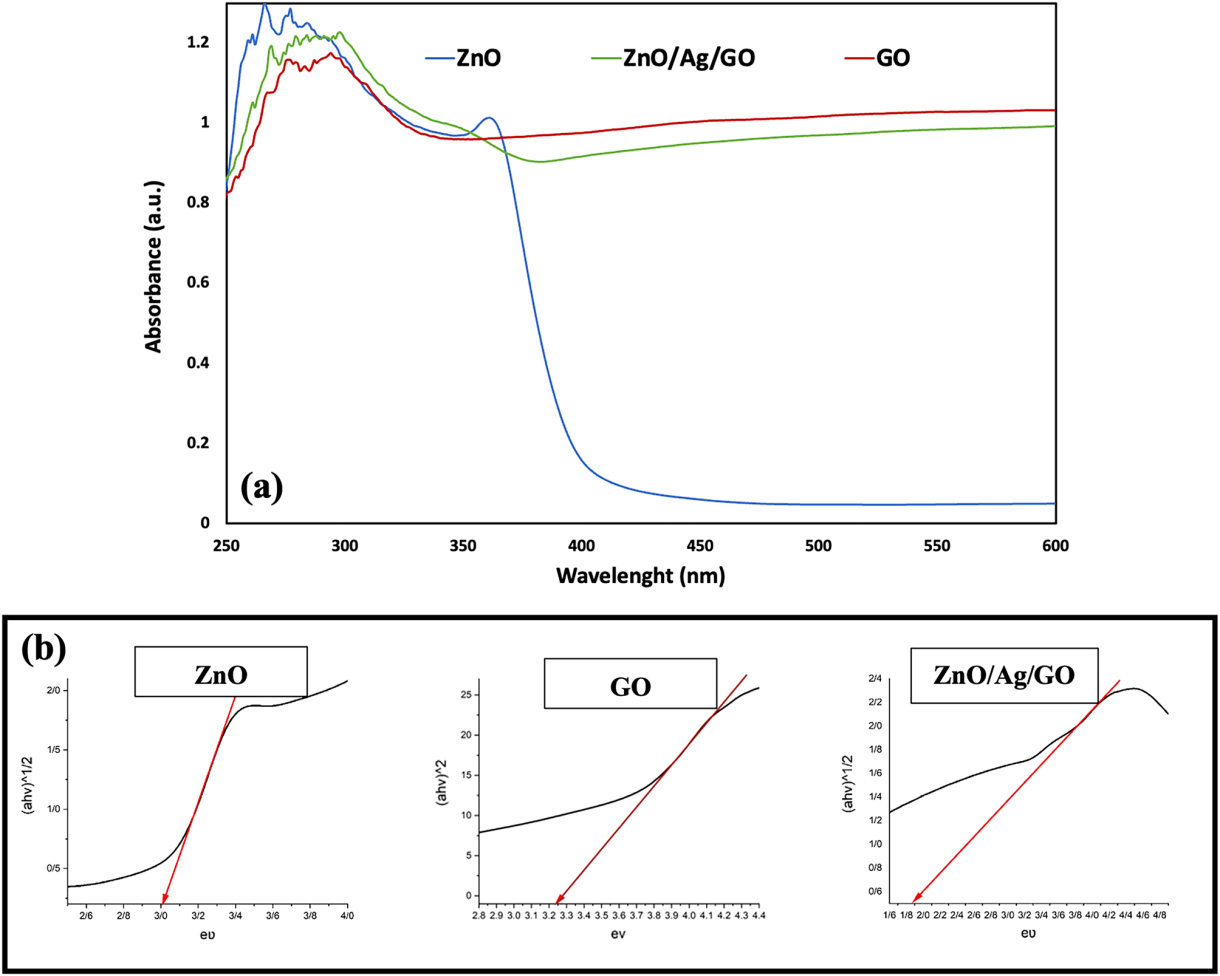
(**a**) DRS results of ZnO NPs, GO and ZnO/Ag/GO powders. (**b**) Tauc diagrams of the powders.

The UV–Vis diffuse reflectance spectroscopy (DRS) technique was performed to find out the effect of making composites on the optic properties of ZnO (Fig. 2). The band gap energy of the samples was also estimated by drawing Tauc diagrams, where x and y axes presented *hν* and (*αhν*)^2^, respectively^34^. While ZnO NPs showed no obvious absorption in the visible region, a strong absorption was seen in the UV region due to their large band gap energy (~ 3 eV). GO exhibited considerable absorption in both the UV and visible regions. Thus, the ZnO/Ag/GO sample also presented a strong absorption in both wavelength regions. Interestingly, because of the synergistic effect, the band gap energy of the composite was significantly lower than that of both ZnO NPs and GO (~ 3.2 eV). Therefore, the ZnO/Ag/GO sample is more prone to electron excitation and transition^35^.

### Antibacterial assessments of the nanostructures

To evaluate the effect of developing ZnO/Ag/GO nanocomposite on the antibacterial activity of ZnO, the MIC and the MBC values of ZnO NPs, GO, ZnO/GO and ZnO/Ag/GO against *S. aureus* and *E. coli* were compared together. The results are shown in Table 1.

**Table 1:** MIC and MBC values of ZnO NPs, GO, ZnO/GO and ZnO/Ag/GO against *S. aureus* and *E. coli.*

Samples	Strains			
	*S. aureus* (ATCC 29,213)		*E. coli* (ATCC 25,922)	
	MIC (µg/ml)	MBC (µg/ml)	MIC (µg/ml)	MBC (µg/ml)
ZnO NPs	1000	> 1000	> 1000	> 1000
GO	1000	> 1000	> 1000	> 1000
ZnO/GO	62	1000	125	> 1000
ZnO/Ag/GO	31	500	62	1000

The antibacterial activities of GO and ZnO nanoparticles are well recognized. Researchers are making serious efforts to improve their antibacterial activity through various strategies, including making Zn- or GO-based nanocomposites. Based on the results, making a composite of ZnO and GO (the ZnO/GO sample) resulted in a substantial decrease in the MIC value compared to ZnO NPs and GO. The incorporation of Ag has improved the antibacterial activity even more. ZnO/Ag/GO presented approximately 30 times more antibacterial effects compared to ZnO NPs. Making a hybrid composite can enhance the activity of GO and ZnO against bacteria in different ways. Besides the synergistic effects, it was reported that the decoration of GO sheets with nanoparticles leads to an improvement in dispersion, roughness and hydrophilicity of GO, resulting in enhanced GO interaction with cells. At the same time, GO sheets are able to improve the stability and optimize the release rate of the non-GO part^35^. Furthermore, the substantially lower band gap energy of the composite can cause a higher level of reactive oxygen species (ROS) in the cells as the dominant mechanism of action of ZnO NPs^19^.

### Morphological evaluation of filter papers

**Figure 3:**
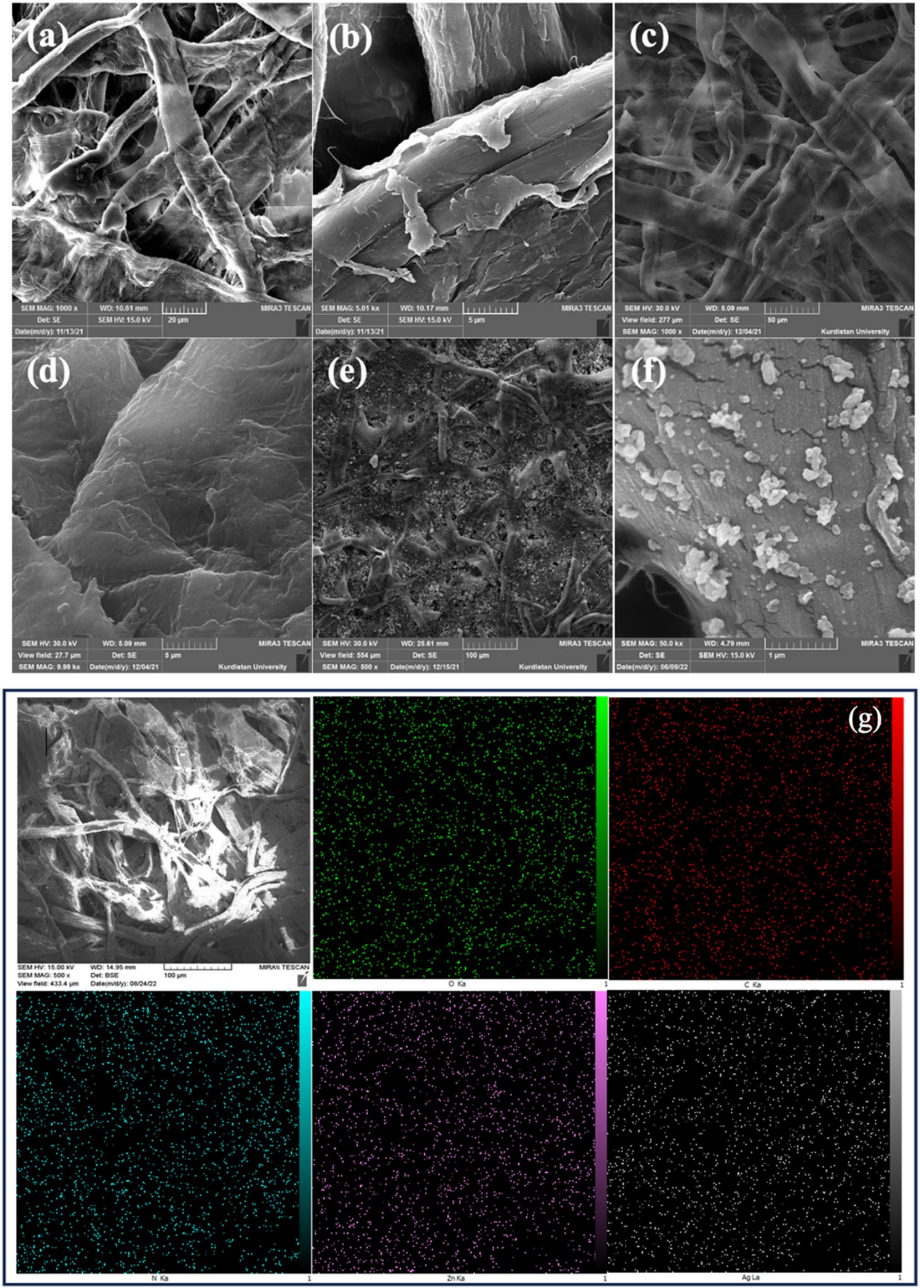
FE-SEM images from the surface of (**a** and **b**) unmodified papers. (**c** and **b**) papers modified with PDA/PEI (cationic papers) and (**e** and **f**) ZnO/Ag/GO@PDA/PEI papers. (**g**) Elemental EDS mapping of ZnO/Ag/GO@PDA/PEI papers.

After the synthesis of the antibacterial ZnO/Ag/GO nanocomposite, the surface of the cellulose filter papers was modified with PDA, PEI and the nanocomposite. Figure 3 shows the FE-SEM images of unmodified papers along with the PDA/PEI modified papers (cationic papers) and ZnO/Ag/GO@PDA/PEI papers.

The cellulose papers consist of microfibers, and the voids between the fibers are visible in the FE-SEM images. The reported mean pore size of the unmodified papers was 2.5 μm. No considerable differences were observed after modification with PDA/PEI. However, the fibers in the PDA/PEI modified papers appear to be more tightly pressed than the unmodified papers, and the surface of the fibers seems smoother. Particles, attached and distributed on the fibers, are visible in ZnO/Ag/GO@PDA/PEI papers. Especially, ZnO/Ag parts can be seen on the surface of the fibers in the image with higher magnification (Fig. 3f). The EDS elemental mapping analysis results are presented in Fig. 3g. The mapping results showed a uniform distribution of nitrogen related to the amine groups of PDA and PEI molecules and Zn and Ag, indicating the successful distribution of the nanocomposite particle throughout the matrix.

Dopamine, as a highly reactive molecule, can form a polycationic PDA layer on organic or inorganic surfaces, including cellulose filter papers, by self-polymerization. The optimum conditions for forming uniform PDA coatings on membranes, e.g., PD concentration and pH, have been the subject of several studies^36^, and the conditions in the present study have been chosen accordingly. In the dopamine-containing Tris buffer, dopamine molecules can react with the hydroxyl groups of cellulose papers via their catechol groups to form stable chemical bonds and subsequently a PDA stacking layer^37^. Dopamine can also oxidize, forming reactive quinone, which is able to form covalent bonds with the amine groups of PEI molecules by Michael addition or Schiff base reaction^17,38^. Moreover, dopamine can react with metal and metal oxide nanoparticles and the surface of functional groups of GO by surface complexation through various routes, such as via catechol, bridge bidentate and hydrogen bonding^39^.

### The zeta potential of filter papers

**Figure 4:**
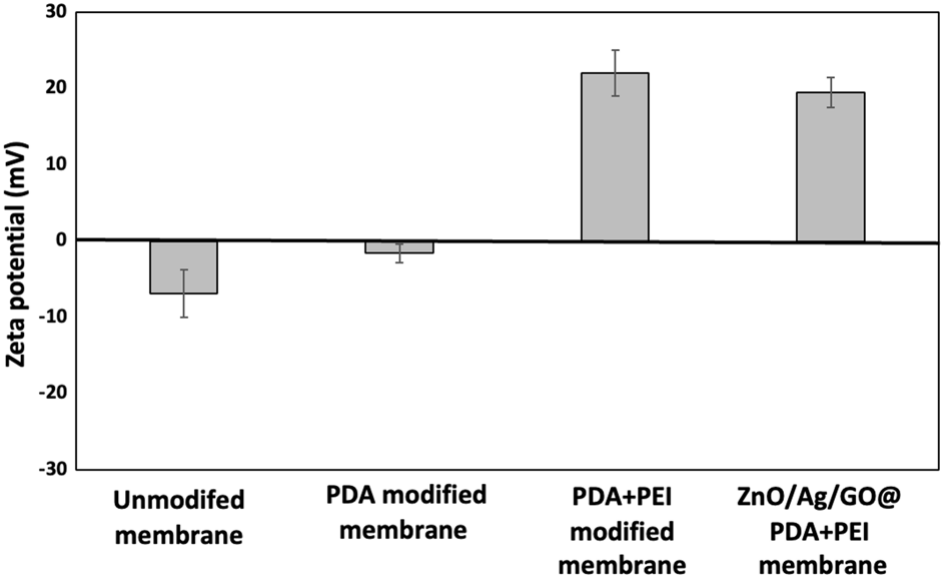
Zeta potential of ZnO/Ag/GO@PDA/PEI PTFE membranes. Error bars exhibit the standard deviation from three calculations.

The measurement of the zeta potential of papers with proper accuracy was not technically possible due to the high water flux rates of the papers. However, Zeta potential estimations were performed using polytetrafluoroethylene (PTFE) MF membranes to provide insight into the surface charge of the modified papers. The same processes were used to produce ZnO/Ag/GO@PDA/PEI PTFE membranes. The FE-SEM images and EDS mapping results of the modified membranes are presented in the supplementary information. The zeta potentials were estimated at pH 7 and are shown in Fig. 4.

The unmodified PTFE membranes represented a negatively charged surface (zeta potential − 6.8 mV). PDA coating resulted in a less negative value (− 1.6 mV). Therefore, the increase in the positive surface charge of the filters after the formation of the PDA layer was small because PDA is amphoteric^40^. However, PDA, thanks to its abundant chemical reaction sites, can function as an intermediate layer to permit the filter to be more modified using other molecules^36^. Thus, PEI was used to form a layer with higher positive charge. PEI due to the presence of many amino groups, including primary, secondary, and tertiary ones along its chain can present a high positive charge density^41^. Performing PEI led to charge reversal and changed the zeta potential to + 22 mV. ZnO/Ag/GO incorporation has slightly reduced the zeta potential and brought it to + 19.5 mV due to the negative zeta potential of the nanocomposite (− 12.7 mV). Therefore, the results of the zeta potential test along with the EDS mapping test verify the presence of cationic functional groups on the surface of the modified papers.

### Flow rates of filter papers

**Figure 5:**
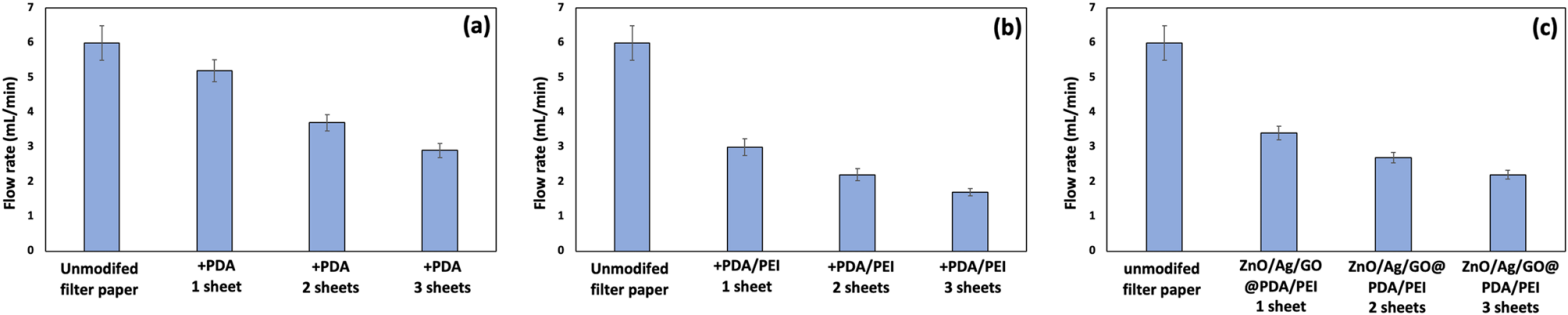
Flow rates for the unmodified and modified papers due to gravity using various numbers of papers. (**a**) PDA modified papers. (**b**) PDA/PEI modified papers. (**c**) ZnO/Ag/GO@PDA/PEI papers. Error bars exhibit the standard deviation from three calculations.

The flow rate of the papers has been investigated to check whether the flow rate, due to gravity, was still acceptable after the modification. The flow rate of the unmodified paper was about 6 mL min^-1^. After modification with PDA (Fig. 5a), no significant difference was seen (5.2 mL min^-1^). For all papers, the flow rate reduced with increasing the number of sheets. The filter with three sheets indicated a flow rate of ~ 3 mL min^-1^. With the incorporation of PEI, the flow rates were affected more (Fig. 5b). Thus, the polymerization of PEI had an impact on the pore size of papers. However, large-pore blockage has not happened, and the filters can still work under atmospheric pressure. Pore blockage and the subsequent significant flux decline after PDA grafting on UF or NF membranes are very probable^36^. For 3 sheets of PDA/PEI modified papers, the flow rate was 1.7 mL min^-1^. The presence of ZnO/Ag/GO on the surface of papers slightly improved the flow rate (Fig. 5c), probably due to the hydrophilicity of nanostructures. The flow rates were 3.4 and 2.2 mL min^-1^ for one and three sheets, respectively. Therefore, despite the reduction in flow rate, the modified filters can still purify water under atmospheric pressure.

### Bacterial removal efficiency of filter papers

**Figure 6:**
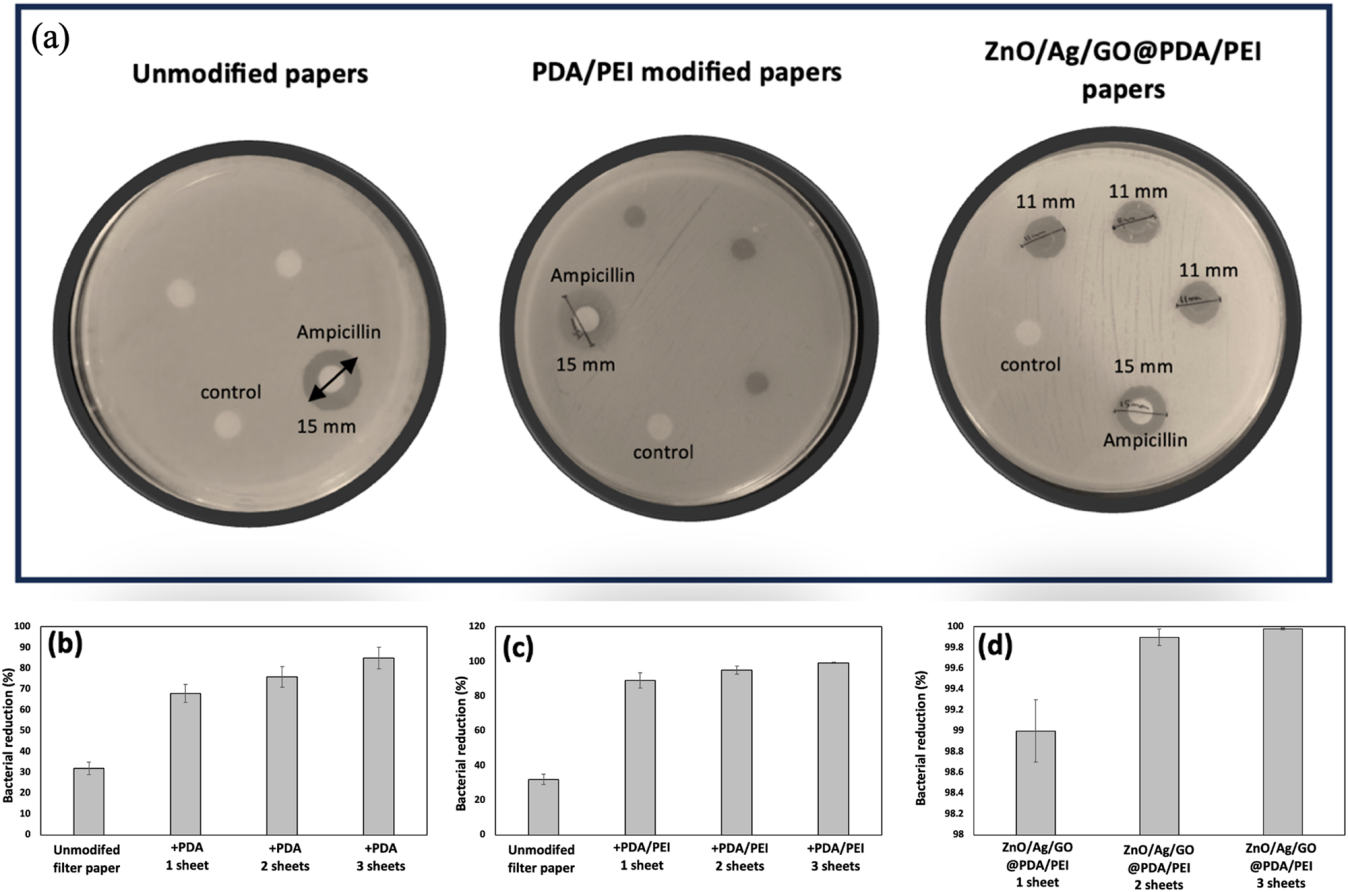
(**a**) Antibacterial assays of the unmodified and modified papers with disk diffusion tests against *E. coli*. Ampicillin and an empty disc were used as positive and negative controls, respectively. Bacterial removal efficiency of the unmodified and modified papers, due to gravity, using various numbers of papers. (**b**) PDA modified papers. (**c**) PDA/PEI modified papers. (**d**) ZnO/Ag/GO@PDA/PEI modified papers. Error bars exhibit the standard deviation from three calculations. Some error bars are too small to be observed.

A disc diffusion test was performed to analyze the antibacterial potential of the modified papers against *E. coli*. As shown in Fig. 6a, the unmodified and PDA/PEI modified papers exhibited no obvious zone of inhibition (ZOI). In contrast, ZnO/Ag/GO@PDA/PEI papers resulted in a ZOI of 11 mm). Furthermore, ampicillin, as the positive control, displayed a ZOI of 15 mm. ZnO, GO and Ag particles cannot diffuse out of the discs in the agar medium and the observed ZOI is only related to the contribution of released soluble species, such as Zn^2+^ and Ag^+^ ions^42^. Thus, the potential of the modified papers as antibacterial filters was concluded.

The bacterial removal efficiency was studied using the filtration of 10 mL of water with a bacterial concentration of 1.5 × 10^8^ CFU/mL and the results are illustrated in Fig. 6. The unmodified paper provided a filtration ability of about 32%. PDA incorporation enhanced the removal efficiency. Moreover, the efficiency improved with increasing the number of sheets performed. One, two and three sheets of PDA modified papers showed bacterial removal efficiency of 68%, 76% and 85%, respectively. PEI incorporation improved the filtration ability and the efficiency of 89%, 95% and 99.2% were achieved using one, two and three sheets of PDA/PEI modified papers, respectively. The increase in bacteria removal efficiency can be justified due to the significant increase in the positive charge on the surface of the papers. However, the role of the PDA/PEI layer was not only to trap bacteria. It has been reported that some cationic polymers, including PDA and PEI, also have antibacterial activity and can destabilize the bacterial membrane. Their activity heavily depends on their structure, such as their molecular weight, type of functional groups, and form (linear or branched)^41,43,44^. Moreover, the combination of those cationic polymers with other antibacterial agents has led to the synergistic improvements in activity^45^. The lowest limit for efficient bacterial filtration ability is more than 99% (2-log reduction), showing the effectiveness of using three sheets of PDA/PEI modified papers. However, the requirement by the World Health Organization (WHO) for *E. coli* removal in a favorably protective household water treatment is the filtration of more than 99.99% of bacteria^8^. The incorporation of ZnO/Ag/GO nanostructures as strong antibacterial agents significantly enhanced the filtration ability. One, two and three sheets of ZnO/Ag/GO@PDA/PEI papers displayed bacterial removal efficiency of 99%, 99.8% and 99.98%, respectively, which met the WHO standards.

### Leaching of nanoparticles

The concentrations of leached zinc and silver in the filtrate were investigated since zinc and silver are potentially harmful to people at high doses. Based on the WHO’s guidelines, 0.1 and 3 ppm were considered the maximum allowed concentrations of Ag and Zn in drinking water, respectively^17,46^. The concentrations of the elements in filtrates after the filtration using different papers, obtained by ICP/MS, are presented in the supplementary information. One of the roles of the formed PDA/PEI layer, besides forming a cationic layer, has been the formation of stable bonds between the nanocomposites and the surface of the papers. Thus, in order to study this matter, ZnO/Ag/GO impregnated papers were also synthesized, in which the nanocomposites were directly attached to the surface of the papers. The modification process of these papers was the same as the modification process of papers without the presence of PDA/PEI. The leached zinc concentration of ZnO/Ag/GO impregnated papers was found to be 2.84 ppm, which was close to the 3 ppm limit. PDA/PEI incorporation significantly reduced the leached zinc concentrations. In the filtrate of three sheets of ZnO/Ag/GO@PDA/PEI papers, the Zn concentration was 380 ppb, which is far below the WHO’s limit. The leached silver concentrations were also much lower than the 100 ppb limit. It is evident that a more stable bond was made between the nanocomposites and the cationic surface layer of the papers compared to the ZnO/Ag/GO impregnated papers.

### Reuse of filter papers

**Figure 7:**
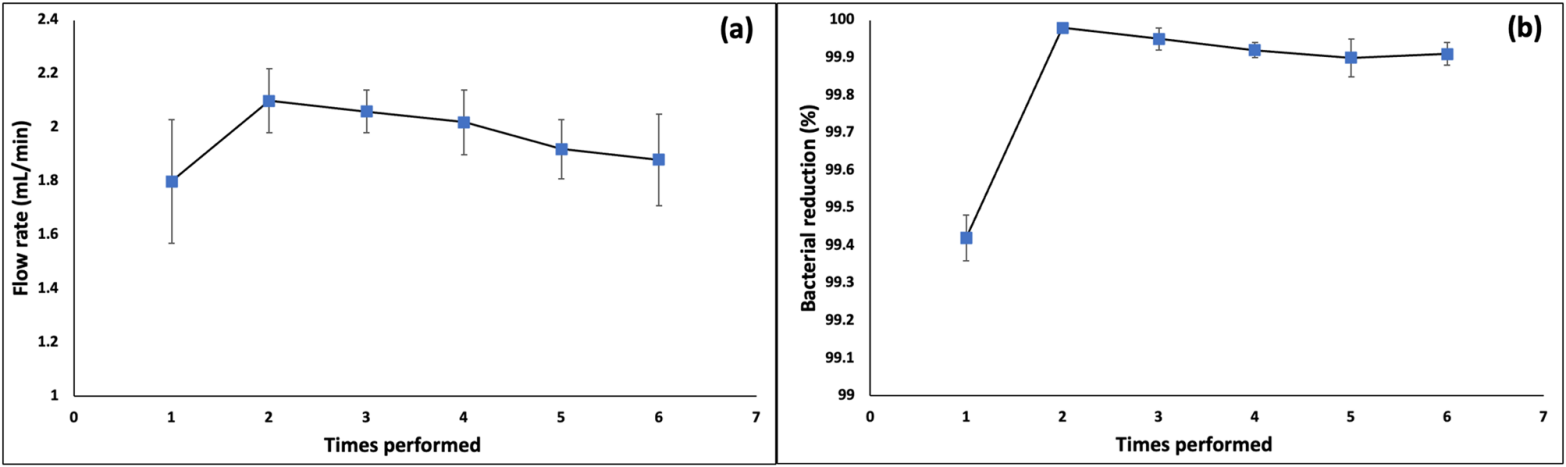
Changes in (**a**) the flow rate and (**b**) the bacterial removal efficiency with the number of times filters were used. All the experiments were done using 3 sheets of ZnO/Ag/GO@PDA/PEI papers. Error bars exhibit the standard deviation from three calculations.

The dependency of the flow rate and the bacterial removal efficiency of the papers on the number of times they were performed was investigated by re-performing the papers for six consecutive filtration procedures. For this assay, three sheets of ZnO/Ag/GO@PDA/PEI papers were chosen, and the results are presented in Fig. 7. In all tests, water with a bacterial concentration of 1.5 × 10^8^ CFU/mL was used. The first filtration presented a somewhat lower flow rate and bacterial removal efficiency than the later filtration procedures, probably because of the dry condition of the papers for the first filtration. The cationic layer needs to be in a wet condition to become completely charged. Moreover, it was reported that the pores may shrink after absorbing water due to a growth in the thickness of the fibers^8^. Accordingly, before performing the papers for the estimation of the flow rate and the bacterial removal efficiency, they were washed with water (described in “Evaluation of nanostructures leaching into the filtered water” and “Bacterial removal efficiency of filtration” sections). The flow rate after the second filtration reduced slightly, maybe due to the blocking of some pores by bacteria. However, the flow rates are still more than 1.8 mL.min^-1^. The bacterial removal efficiency was more than 99.9% for the 2nd to 6th filtration processes. Therefore, the papers can be re-performed multiple times with acceptable flow rates and bacterial removal performances.

### Comparison of the results with the reported literature

The use of filters with large pores to remove bacteria has been investigated not only to supply drinking water but also in the food and medical industries^47^. Most of the studies have been related to the use of POU ceramic filters compared to polymeric or cellulose paper filters^48^. However, their high cost restricts their applications^25^. Cellulose, as the most abundant polysaccharide on earth, has been considered a cost-effective and eco-friendly material for use in different applications^49^. As cellulose-based filters are performed in disposable everyday filters, such as tea, coffee and dust filters.

Ghodake and et al. embedded silver nanoparticles onto PP/PE fabrics for the layer-by-layer (LBL) modification of cellulose filters, and an *E. coli* removal efficiency of 99.4% was achieved^50^. Though, no results have been reported regarding the effect of surface modification on the flow rate. The LBL technique was also used by Ottenhall et al., in which the LBL assembly was used by polyelectrolytes to develop cellulose-based filters^8^. The bacteria-reducing efficiency was more than 99.9% when twenty sheets of three-layer LBL filters were used. Compared to the present study, relatively the same trend has been observed regarding the effect of the surface modification and the number of sheets on the flow rate. However, the use of 10 sheets of filters has led to about an 80% reduction in flow rate. Furthermore, one drawback of the LBL technique is the relatively long time needed to assemble layers^51^. Chien et al. have successfully modified the surface of commercial cellulose filter papers with PDA/PEI and silver nanoparticles^17^. Only a 10% decline in pure water flux was observed, but ‌the bacterial removal was ~ 99%. Coating cellulose filter papers with Ag nanoparticles was also reported in other studies. Praveena et al. have reduced silver nitrate using sodium borohydride to graft silver nanoparticles on cellulose filter papers to remove *E. coli* from water^52^. Polycationic polymers decorated with Ag nanoparticles were also performed to modify polymeric MF membranes for pathogen removal^16^.

As stated previously, compared to those studies, we have developed filter papers that remove bacteria to the same or greater extent while much cheaper nanoparticles than silver nanoparticles have been used. Zinc nitrate as a precursor for the synthesis of ZnO nanostructures is about 20 times cheaper than silver nitrate. Moreover, the cytotoxicity of zinc oxide is much lower than that of silver^53^.

## Conclusions

In this study, commercial cellulose filter papers were successfully modified using immobilizing PDA/PEI and ZnO/Ag/GO nanostructures as cationic polymers and antibacterial agents, respectively, on the surface of the papers for bacterial removal from water with no need for energy. The filter papers were engineered to remove bacteria by the entrapment of them via the cationic surface layer, and the antibacterial activity of the nanostructures. We used ZnO-based nanocomposites because they presented unique potential to address the current challenges of using membranes in water and wastewater treatment. The developed ZnO/Ag/GO nanostructures exhibited strong antibacterial activity, with approximately 30 times more antibacterial effects compared to ZnO NPs due to the synergistic effects. Although the modification leads to a relative decrease in the flow rate of filter papers (~ 40% reduction), the modified ones have the ability to remove about 99% of bacteria under atmospheric pressure. Stacking three sheets of them can improve bacterial reduction by up to about 99.99%, which meets the WHO standards. We indicated that the modified papers can be reused multiple times with a suitable flow rate and bacterial removal performance. The bacterial removal efficiency was more than 99.9% after the 6^th^ filtration process, while only a 20% reduction in flow rate has occurred. Additionally, the papers were engineered in such a way that no leaching of nanostructures in the filtrate happened. The developed papers can instantly treat water on site, and the simplicity of their use is a favorable way to supply safe water for people suffering from a lack of access to drinking water, which is estimated to be around one billion globally. The assessments for studying the potential of the papers for other pathogen removal, such as viruses, are presently in progress at our lab. Moreover, we are working on the development of gravity-driven polymeric membranes using the same strategy to disinfect water for long periods of time, such as years, without the need for complex maintenance.

### Supplementary Information

Supplementary Information.

## Data Availability

The datasets used and/or analysed during the current study available from the corresponding author on reasonable request.
