# Supplementary material for: Cationic cellulose filter papers modified with ZnO/Ag/GO nanocomposite as point of use gravity-driven filters for bacterial removal from water
Source: Sci Rep. 2023 Dec 18;13:22604. doi: 10.1038/s41598-023-50114-3 (PMC10730911; doi:10.1038/s41598-023-50114-3)
Supplement: Supplementary file 1 — Supplementary Information. [file 41598_2023_50114_MOESM1_ESM.pdf]

Supplementary information

Cationic cellulose filter papers modified with ZnO/Ag/GO nanocomposite as point of use gravity-driven filters for bacterial removal from water

Seyed-Behnam Ghaffari[a], Mohammad-Hossein Sarrafzadeh[a*]

[a] UNESCO Chair on Water Reuse, School of Chemical Engineering, College of Engineering, University of Tehran, Tehran, Iran.

1. The morphology of pristine and the modified PTFE MF membranes

Polytetrafluoroethylene (PTFE) MF membranes were bought from the FiltraTECH company. The reported size of PTFE membranes was 450 nm. FE-SEM was performed to study the surface of the pristine and modified PTFE membranes, and the results are shown in Fig. 1. Pristine PTFE membranes are composed of nanofibers, bead-like particles and nods. After the PDA/PEI modification, it seems that the decrease in membrane pore size was insignificant. After nanocomposite incorporation, ZnO/Ag/GO particles are uniformly scattered on the surface of the membranes. Additionally, large holes are still visible, and no large pore blockages were seen. The EDS elemental mapping analysis results are presented in Fig. 2. The mapping shows the uniform distribution of nitrogen, zinc and silver, confirming the presence of PDA/PEI and the ZnO/Ag/GO particles.

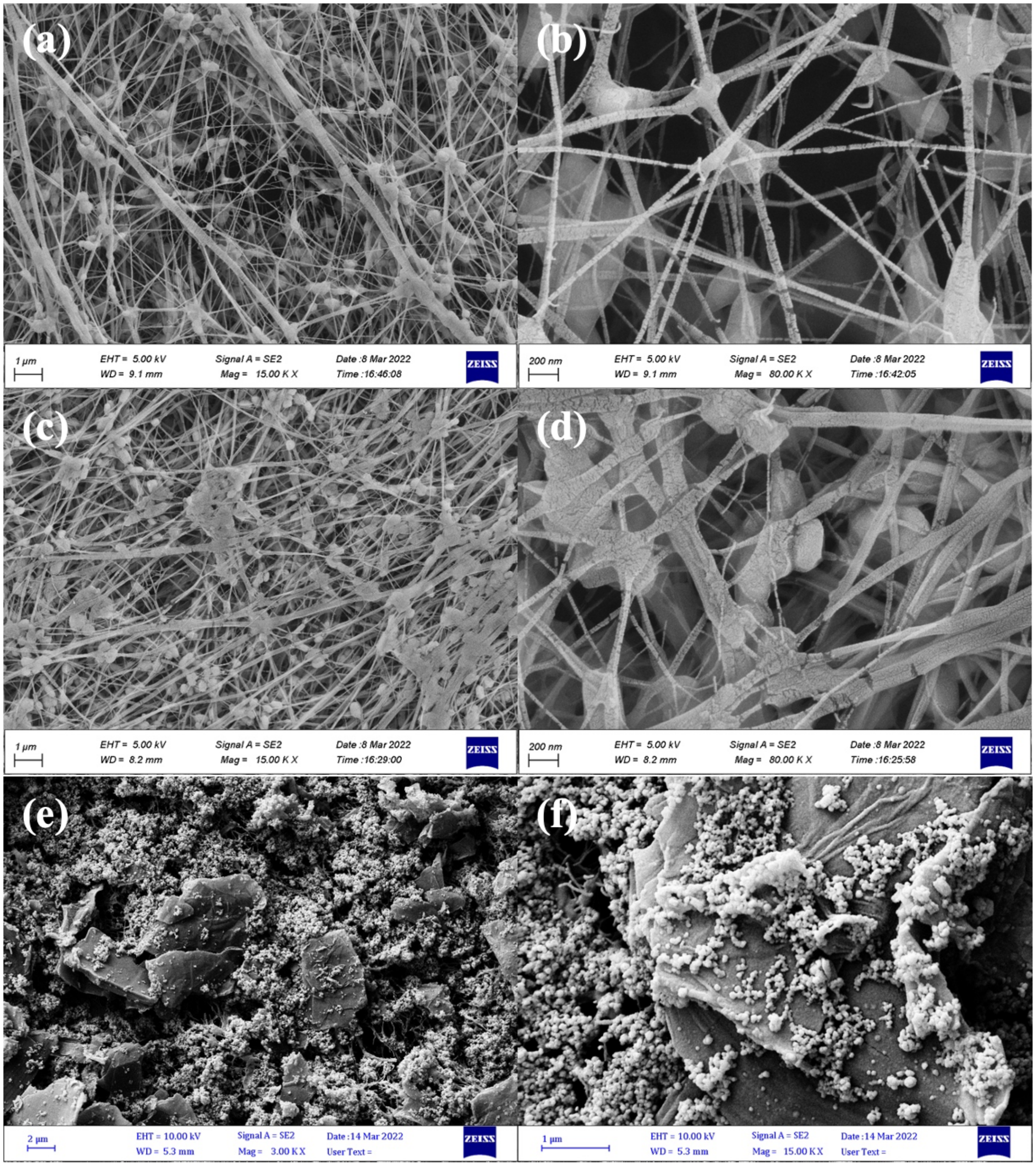

Fig. 1 The FE-SEM images from the surface of (a and b) pristine PTFE membrane. (c and b) membranes modified with PDA/PEI and (e and f) ZnO/Ag/GO@PDA/PEI PTFE membranes.

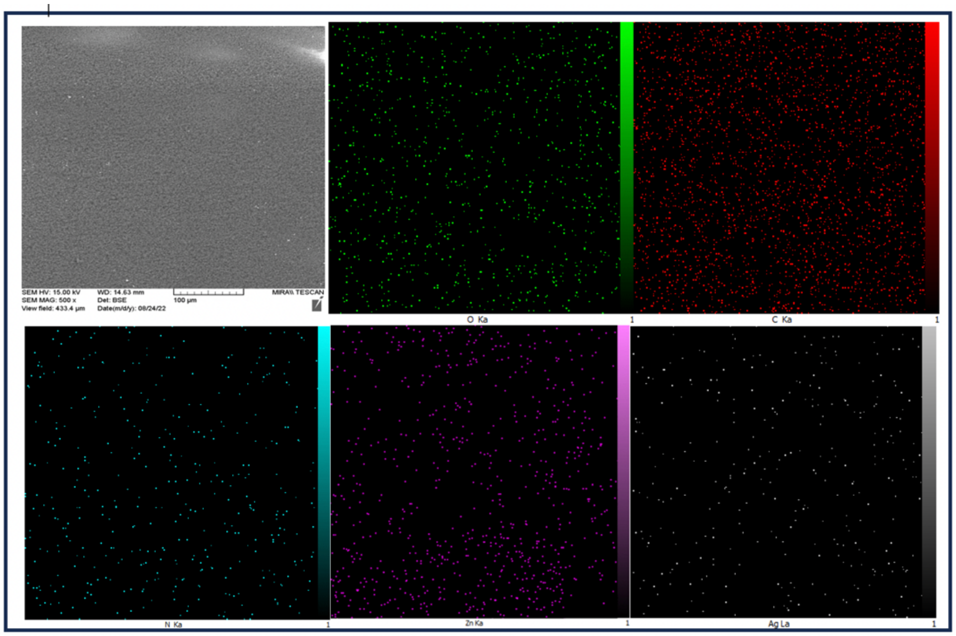

Fig. 2 Elemental EDS mapping of the ZnO/Ag/GO@PDA/PEI PTFE membrane.

2. The concentrations of leached zinc and silver in the filtrate

The concentrations of Zn and Ag in filtrates after the filtration using different papers are presented in Table 1.

Table 1. The leached zinc and silver concentrations in the filtrate after the filtration with different papers, obtained using ICP/MS.

sample	Zn concentration (ppm)	Silver concentration (ppm)
ZnO/Ag/GO impregnated papers, 1 sheet	2.84	0.38
ZnO/Ag/GO@PDA/PEI papers, 1 sheet	0.22	<0.01
ZnO/Ag/GO@PDA/PEI papers, 2 sheets	0.31	<0.01
ZnO/Ag/GO@PDA/PEI papers, 3 sheets	0.38	0.01
